# Identification and Classification of Alzheimer’s Disease Patients Using Novel Fractional Motion Model

**DOI:** 10.3389/fnins.2020.00767

**Published:** 2020-09-17

**Authors:** Lei Du, Boyan Xu, Zifang Zhao, Xiaowei Han, Wenwen Gao, Sumin Shi, Xiuxiu Liu, Yue Chen, Yige Wang, Shilong Sun, Lu Zhang, Jiahong Gao, Guolin Ma

**Affiliations:** ^1^Department of Radiology, China-Japan Friendship Hospital, Beijing, China; ^2^Graduate School of Peking Union Medical College, Chinese Academy of Medical Sciences and Peking Union Medical College, Beijing, China; ^3^Beijing Intelligent Brain Cloud Inc., Beijing, China; ^4^Department of Anesthesiology, Peking University First Hospital, Peking University, Beijing, China; ^5^Department of Science and Education, Shangluo Central Hospital, Shangluo, China; ^6^Beijing City Key Lab for Medical Physics and Engineering, Institute of Heavy Ion Physics, School of Physics, Peking University, Beijing, China; ^7^Center for MRI Research, Academy for Advanced Interdisciplinary Studies, Peking University, Beijing, China; ^8^McGovern Institute for Brain Research, Peking University, Beijing, China

**Keywords:** diffusion magnetic resonance imaging, fractional motion model, anomalous diffusion, Alzheimer’s disease, hippocampus

## Abstract

Most diffusion magnetic resonance imaging (dMRI) techniques use the mono-exponential model to describe the diffusion process of water in the brain. However, the observed dMRI signal decay curve deviates from the mono-exponential form. To solve this problem, the fractional motion (FM) model has been developed, which is regarded as a more appropriate model for describing the complex diffusion process in brain tissue. It is still unclear in the identification and classification of Alzheimer’s disease (AD) patients using the FM model. The purpose of this study was to investigate the potential feasibility of FM model for differentiating AD patients from healthy controls and grading patients with AD. Twenty-four patients with AD and 11 healthy controls were included. The left and right hippocampus were selected as regions of interest (ROIs). The apparent diffusion coefficient (ADC) values and FM-related parameters, including the Noah exponent (α), the Hurst exponent (*H*), and the memory parameter (*μ*=*H*−1/*α*), were calculated and compared between AD patients and healthy controls and between mild AD and moderate AD patients using a two-sample *t*-test. The correlations between FM-related parameters α, *H*, μ, and ADC values and the cognitive functions assessed by mini-mental state examination (MMSE) and Montreal cognitive assessment (MoCA) scales were investigated using Pearson partial correlation analysis in patients with AD. The receiver-operating characteristic analysis was used to assess the differential performance. We found that the FM-related parameter α could be used to distinguish AD patients from healthy controls (*P* < 0.05) with greater sensitivity and specificity (left ROI, 0.917 and 0.636; right ROI, 0.917 and 0.727) and grade AD patients (*P* < 0.05) showed higher sensitivity and specificity (right ROI, 0.917, 0.75). The α was found to be positively correlated with MMSE (*P* < 0.05) and MoCA (*P* < 0.05) scores in patients with AD, indicating that the α values in the bilateral hippocampus were a potential MRI-based biomarker of disease severity in AD patients. This novel diffusion model may be useful for further understanding neuropathologic changes in patients with AD.

## Introduction

Alzheimer’s disease (AD) is the most common neurodegenerative disease and is characterized by memory loss and cognitive decline (Reddy and Oliver, 2019). According to the World Health Organization (WHO), dementia affects nearly 47.5 million individuals worldwide and still increases by approximately 7.7 million new cases each year (Khan et al., 2017; Salvatore et al., 2018). As the most common type of dementia, AD may account for 60–70% of these cases (Wortmann, 2012; Alzheimer’s Association, 2016; Khan et al., 2017) and has a significant impact on the life quality of patients and societal costs. The pathogenesis of AD is extremely complicated, mainly including the deposition of amyloid-β (Aβ) and hyperphosphorylation of tau protein, which results in the formation of Aβ-plaques and intracellular neurofibrillary tangles (NFTs) separately (Kidd, 1963; Hyman et al., 1984; Braak and Braak, 1991; Wegmann et al., 2010; Aisen et al., 2017), then causes neuronal death. Now the diagnosis of AD is complicated and the accuracy is difficult. Therefore, it is of great significance to develop an effective diagnostic method for AD in clinical research (Cummings, 2017).

Diffusion magnetic resonance imaging (dMRI) is a powerful and non-invasive tool that can describe the random motion of water molecules in biological tissues, provide unique information about the microscopic properties, and is highly sensitive to detecting changes in gray and white matter in the brain (Yoshiura et al., 2003). The diffusion process of water molecules in the nervous system is directionally dependent (Chenevert et al., 1990; Moseley et al., 1990). And this directional dependence, namely anisotropy, occurs primarily due to the inherent axonal membranes that hamper water molecule diffusion and the dense packing of axons (Beaulieu, 2002). Measurements of the anisotropy of water molecules diffusion at the micron level reflect the changes of the underlying microstructure.

Compared with other magnetic resonance imaging (MRI) modalities, dMRI investigates the diffusion process at the cellular scale in tissues (e.g., micrometers), which is far superior to the typical millimetric image resolution (Le Bihan and Johansen-Berg, 2012). At present, one of the most widely used dMRI techniques in clinical application is the apparent diffusion coefficient (ADC), and *b*-values obtained by dMRI frequently range from 0 to 1000 s/mm^2^. Apparent diffusion coefficient is widely used in clinical practice, and it can be used to differentiate brain tumors (Yamasaki et al., 2005) and grade tumors (Bulakbasi et al., 2004), and distinguish mild cognitive impairment (MCI) and AD (Pietroboni et al., 2018; Xue et al., 2019). Diffusion tensor imaging (DTI) is another widely used dMRI technique in the research. The fractional anisotropy (FA) and mean diffusivity (MD) obtained from DTI are potential biomarkers of brain abnormalities in patients with MCI and AD (Bosch et al., 2012; Nir et al., 2013; Mayo et al., 2018). However, both ADC and DTI have some limitations. Firstly, it is well known that conventional dMRI uses a mono-exponential model and assumes a standard diffusion process in biological tissues. However, many studies have found that the observed dMRI signal decay curve deviates from the mono-exponential form (De Santis et al., 2011). Both ADC and DTI are based on the standard mono-exponential diffusion model, and the observed diffusion-time reflects the non-Gaussian nature of diffusion (Fieremans et al., 2016; Xu et al., 2017a). Secondly, the tensor model of DTI is too simple, which means that its indices can be affected by several features of the microstructure. To solve this problem, many models based on different theories and anomalous diffusion processes have been developed to find the optimal agreement between the experimentally observed signal decay curve and the proposed fitting curves (Mulkern et al., 1999; Maier et al., 2001; Jensen et al., 2005; Magin et al., 2008). These models guarantee a more detailed detection of the differences between disease types and disease grades but their signal decay curves are still different.

Recently, a novel fractional motion (FM) model was developed by *the Center for MRI Research at Peking University.* The FM model is regarded as a more appropriate model in the biophysics community for describing the complex diffusion process in biological systems (Magdziarz et al., 2009; Burnecki and Weron, 2010; Weiss, 2013), and it is a promising model for describing the diffusion process of brain tissue. Several studies have demonstrated that the FM model is a better model to explain the diffusion process of biological living cells (Magdziarz et al., 2009). The FM model assumes that the diffusion process of tissues is *H*-self-similar, α-stable, and has stationary increments (Xu et al., 2017b). The symbol α is the Noah exponent that can quantify the fluctuations of the random process. When α = 2, the increments are Gaussian distributed, while when 0 < α < 2, the increments are Lévy distributed (Xu et al., 2017b; Xu et al., 2017a). *H* is the Hurst exponent, which depicts the self-similarity property of molecular trajectories. μ is the memory parameter and *μ* = *H*−1/*α*. When μ > 0, the increments are positively correlated and show long-range dependence (long memory, persistence), while when μ < 0, the increments of the process are negatively correlated and show short-range dependence (short memory, anti-persistence) (Xu et al., 2017a,b).

Previous studies have found that the FM-related parameter maps of healthy people showed obvious contrasts among normal brain tissues (Fan and Gao, 2015), and the FM-related parameter maps are superior to ADC in differentiating between low-grade and high-grade gliomas (Xu et al., 2017b, 2018). However, the parameters (α, *H*, and μ) of the FM model in identifying AD patients from healthy controls and grading AD patients is still not clear. Therefore, the purpose of this study was to investigate the potential feasibility of the FM model for distinguishing AD patients from healthy controls and grading AD patients.

## Materials and Methods

### Subjects

This study was approved by the ethics committee of China-Japan Friendship Hospital and informed consent was obtained from all subjects. The cognitive function of all participants was assessed using the mini-mental state examination (MMSE) scale and Montreal cognitive assessment (MoCA) scale. A total of 24 AD patients and 11 healthy controls underwent MRI examination and MMSE and MoCA scale assessment. These AD patients visited China-Japan Friendship Hospital between November 2015 and March 2019. The clinical diagnosis of AD met criteria as determined by the National Institute of Neurological and Communicative Disorders and Stroke and the Alzheimer’s Disease and Related Disorders Association (NINCDS-ADRDA) (1984) (Du et al., 2018). Patients met the following criteria: (a) The MR image quality was good, no artifacts; (b) patients had no concurrent brain diseases; and (c) patients had no limb activity disorders, aphasia, visual, and hearing impairment. Healthy controls were recruited from the local community. Healthy controls with a history of cardiovascular, neurologic, metabolic, and psychiatric disorders or brain abnormalities detected by conventional MRI were excluded from this study, and the MMSE scores of healthy control were between 26 and 30. Table 1 shows their clinical characteristics. All participants underwent conventional MRI, 3D T1weighted imaging, and dMRI.

**TABLE 1:** Demographic information and clinical assessment scores for all subjects.

	**AD patients**			
			**Healthy**	***P*-value**
	**Mild AD**	**Moderate AD**	**controls**	
Number	12	12	11	–
Male/female	6/6	3/9	2/9	>0.05
Age	65.83 ± 10.06	72.08 ± 3.75	65.27 ± 6.60	>0.05
Education	13.42 ± 3.06	10.50 ± 3.87	10.64 ± 3.30	>0.05
MMSE score	23.17 ± 11.27	19.08 ± 1.44	28.82 ± 1.08	<0.05
MoCA score	19.50 ± 2.39	16.50 ± 2.15	–	–

*AD, Alzheimer’s disease; MMSE, mini-mental state examination; MoCA, Montreal cognitive assessment.*

### Image Acquisition

Brain MR imaging was acquired on a 3.0 Tesla (T) MRI scanner (GE Healthcare, Discovery MR750, United States) with an eight-channel head coil. Using a special Stejskal-Tanner single-shot spin-echo echo-planar-imaging sequence to obtain dMRI images of all subjects.

We did not fix the diffusion gradient separation time (Δ) in the process of scanning the conventional dMRI sequence in order to fit the FM model. Specifically, Δ was arrayed at 27.060, 39.560, and 52.060 ms. The diffusion gradient amplitude (G_0_) was arranged as 15.67, 19.68, 24.73, 31.06, 39.01, and 49.00 mT/m for each Δ value, which were selected to be approximately evenly spaced on the log axis. The gradient duration constant (δ) was fixed at 20.676 ms. So, we obtained 18 non-zero b-values (151, 239, 377, 595, 939, 1481 s/mm^2^ for Δ at 27.060 ms, 245, 387, 611, 964, 1521, 2399 s/mm^2^ for Δ at 39.560 ms, and 339, 535, 845, 1333, 2103, and 3317 s/mm^2^ for Δ at 52.060 ms) in each gradient direction, respectively. To minimize the effect of diffusion anisotropy, we applied the diffusion gradients in three orthogonal directions (the *x*-axis, *y*-axis, and *z*-axis). Moreover, a total of 12 images with *b* = 0 were obtained.

The dMRI sequence parameters were repetition time (TR) = 3800 ms; echo time (TE) = 110 ms; flip angle (FA) = 90°; number of excitations = 2; accelerating factor = 2; field-of-view (FOV) = 240 mm × 240 mm; matrix size = 128 × 128; slice thickness = 5.0 mm; number of slices = 27; and voxel size = 1.875 mm × 1.875 mm × 5 mm. Since high in-plane resolution was preferable, a large slice thickness had to be chosen in order to achieve an adequate SNR. The total scan time was 8 min 33 s. A T1w MRI was acquired in sagittal plane and the parameters were TR = 6.7 ms; TE = Min Full; FA = 12°; slice thickness = 1.0 mm; number of slices = 192; FOV = 256 mm × 256 mm; matrix size = 256 × 256; and scan time = 4 min 10 s.

### Image Segmentation

**FIGURE 1:**
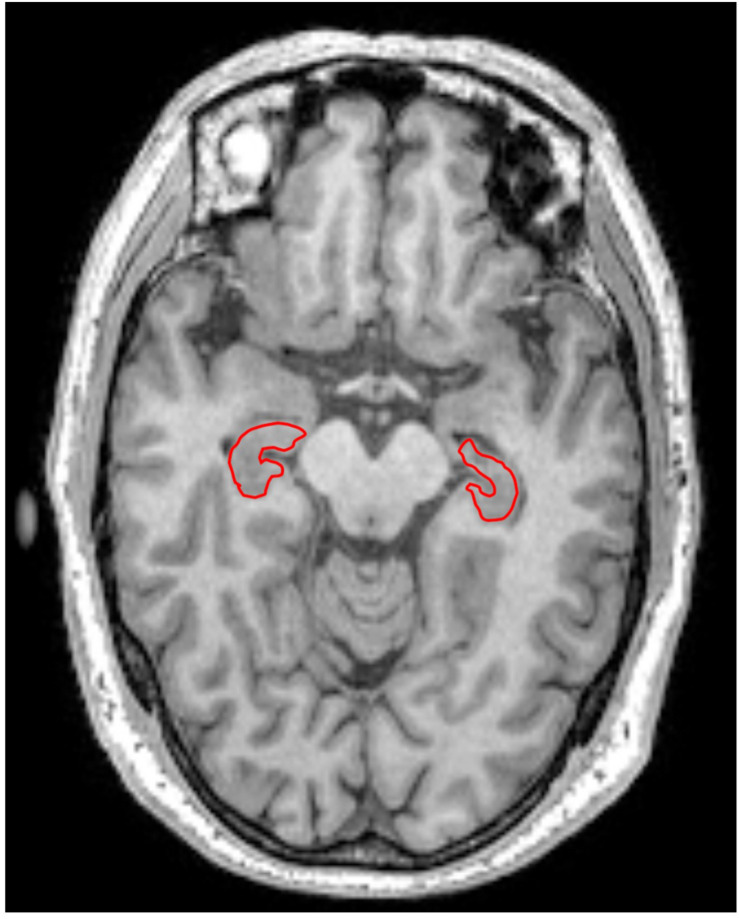
Left and right hippocampus were selected as regions of interest (ROIs) in this study. ROIs were encircled in red line in T1 weighted imaging.

The left and right hippocampus were selected as the regions of interest (ROIs) in the present study (Figure 1). Firstly, the ROIs were drawn manually by an experienced radiologist (Lei Du, 5 years working experience) on the 3D T1 weighted images using MRICRON, then the ROI on T1w MRI was co-registered to diffusion MR imaging to improve the accuracy of the hippocampal outline. In all subjects, the ROIs’ location was segmented and excluded ambiguous voxels. Then the mean values of α, *H*, μ, and ADC in the bilateral hippocampus were acquired.

### Image Analysis

FSL tools were used to correct head motion and eddy current distortions of the obtained images (Cha, 2006). Apparent diffusion coefficient maps were calculated using the images acquired at *b*-values of 0 and 954 s/mm^2^. The images were analyzed using the FM model in order to estimate the anomalous diffusion parameters. According to the dMRI theory based on the FM model (Sui et al., 2015), the diffusion-induced signal decay can be calculated as

(1) $$S/S_{0}=\exp\left(-\eta \,D_{\alpha ,H}\,\gamma^{\alpha }\,G_{0}^{\alpha }\,\Delta^{\alpha +\alpha \,H}\right)$$

where *D*_*α*, H_ refers to the diffusion coefficient of anomalous diffusion, and γ refers to the gyromagnetic ratio. *G*_0_ refers to the diffusion gradient amplitude, and Δ refers to the gradient separation time. η refers to a dimensionless number, which is determined by α, *H*, δ, and Δ (Xu et al., 2017b, 2018). All fitting procedures were carried out by the trust-region-reflective nonlinear fitting algorithm in MATLAB (MathWorks, Natick, MA).

### Statistical Analysis

The age, education, and MMSE score were compared using One-Way ANOVA among mild AD patients, moderate AD patients, and healthy controls. And their data were shown as mean ± SD. Gender was compared using the Chi-square (χ^2^) test. *P* values <0.05 were considered statistically significant.

To investigate the potential feasibility of the FM model for distinguishing AD patients from healthy controls, the mean values of α, *H*, μ, and ADC were compared between AD patients and healthy controls using a two-sample *t*-test. Then these values were also used to identify mild AD and moderate AD patients using a two-sample *t*-test. Besides, in order to quantify the sensitivity and specificity of α, *H*, μ, and ADC values in differentiating patients with AD from healthy controls and distinguishing mild AD patients and moderate AD patients, we generated receiver-operating characteristic (ROC) curves and assessed their area under the curve (AUC). The correlations between α, *H*, μ, and ADC values and the cognitive functions evaluated by the MMSE and MoCA scales were investigated using Pearson partial correlation analysis in patients with AD.

## Results

### Characteristics of All the Subjects

The demographic and clinical test results of all participants are summarized in Table 1. A total of 24 AD patients (9 males and 15 females, mean age 68.96 ± 8.08 years) and 11 healthy controls (2 males and 9 females, mean age 65.27 ± 6.60 years, range 54–78 years, education 10.64 ± 3.30 years) were included in this study. According to the MMSE score and education level, AD patients were divided into a mild AD group (6 males and 6 females, mean age 65.83 ± 10.06 years, range 50–77 years, education 13.42 ± 3.06 years) and a moderate AD group (3 males and 9 females, mean age 72.08 ± 3.75 years, range 67–79 years, education 10.50 ± 3.87 years). There was no significant difference in the age and education among the three groups (*P* > 0.05). While there was a significant difference in the MMSE score among the three groups (*P* < 0.05). And there was a significant difference in the MoCA score between the mild AD group and the moderate AD group (*P* < 0.05) since a MoCA scale was not collected in the healthy group.

**FIGURE 2:**
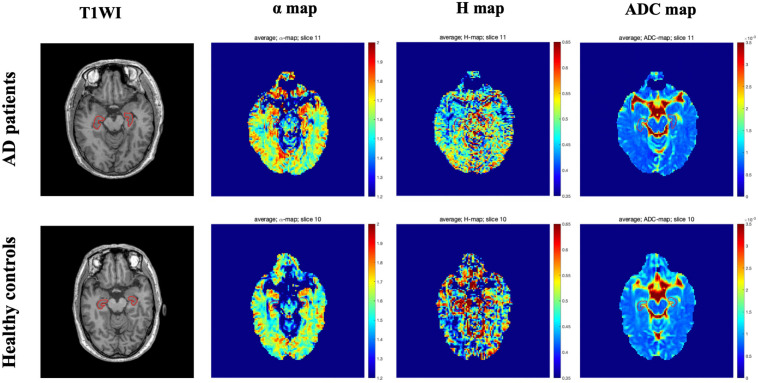
Representative axial T1 weighted imaging and FM-related parameters maps from one AD patient (top row, a 58-year-old male) and one healthy control (bottom row, a 60-year-old male). The bilateral hippocampus are shown with red outlines in all maps. FM, fractional motion; AD, Alzheimer’s disease.

Figure 1 showed the bilateral hippocampus in Axial MRI. Representative maps of the AD group and control group are shown in Figure 2, showing the α, *H*, and ADC maps and T1-weighted images. We can see that there were no obvious image contrasts visible by the naked eye in the α, *H*, and ADC maps in the bilateral hippocampus.

### Comparisons of FM-Related Parameters and ADC Values Between AD Patients and Healthy Controls, and Between Mild and Moderate AD Patients

**FIGURE 3:**
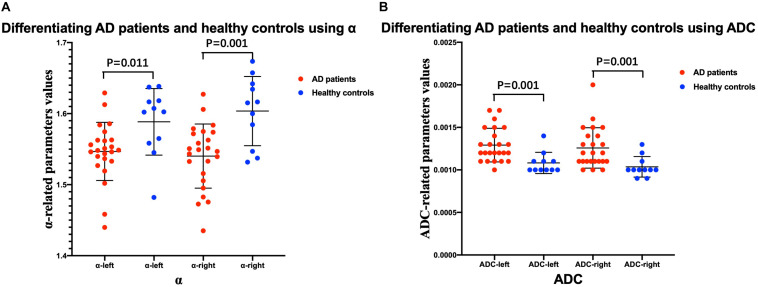
Comparison between AD patients and healthy controls. Scatter plots show that α **(A)** and ADC **(B)** values can readily separate the patients with AD and healthy controls. *n* = 24 for AD patients and *n* = 11 for healthy controls. Two-sample *t*-test was conducted, and *P* < 0.05 was considered as significant. AD, Alzheimer’s disease; ADC, apparent diffusion coefficient.

**FIGURE 4:**
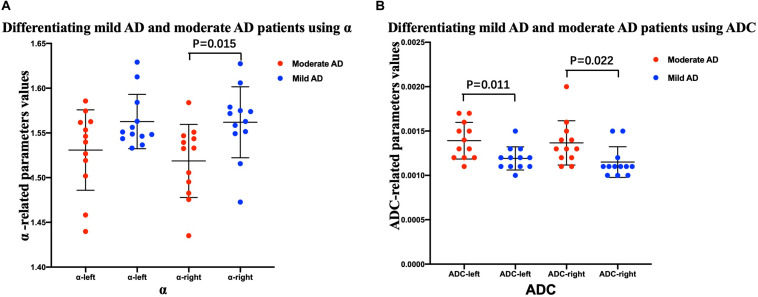
Comparison between mild and moderate AD patients. Scatter plots show that α **(A)** and ADC **(B)** values can readily separate the mild AD and moderate AD patients. *n* = 12 for mild AD patients and *n* = 12 for moderate AD patients. *n* = 24 for AD patients and *n* = 11 for healthy controls. Two-sample *t*-test was conducted, and *P* < 0.05 was considered as significant. AD, Alzheimer’s disease; ADC, apparent diffusion coefficient.

The mean ± SD values of the FM-related parameters and ADC values of the bilateral hippocampus in all subjects are summarized in Table 2. Figures 3, 4 show scatter plots to distinguish AD patients from healthy controls and to differentiate mild AD and moderate AD patients, separately. As shown in Figure 3, the AD patients and healthy controls can be readily separated using α (left ROI, *P*-value = 0.011; right ROI, *P*-value = 0.001) and ADC (left ROI, *P*-value = 0.001; right ROI, P-value = 0.001). Moreover, Figure 4 also shows the identification between mild and moderate AD patients based on FM-related parameters α (right ROI, *P*-value = 0.015) and ADC (left ROI, *P*-value = 0.011; right ROI, *P*-value = 0.022), whereas *H* and μ (both *P*-value > 0.05) failed to differentiate the two groups.

**TABLE 2:** Mean and SD of the FM-related parameters and ADC values of bilateral hippocampus in mild and moderate AD patients and healthy controls.

**Subject**	**No.**	**ROIs**	**FM-related parameters**			**ADC**
			**α**	**H**	**μ**	
Controls	11	Left-hippocampus	1.588 ± 0.047	0.464 ± 0.033	−0.166 ± 0.024	0.00108 ± 0.00013
		Right-hippocampus	1.604 ± 0.049	0.479 ± 0.028	−0.145 ± 0.027	0.00104 ± 0.00012
Mild AD	12	Left-hippocampus	1.563 ± 0.030	0.470 ± 0.027	−0.170 ± 0.031	0.00119 ± 0.00013
		Right-hippocampus	1.562 ± 0.040	0.485 ± 0.040	−0.156 ± 0.045	0.00115 ± 0.00017
Moderate AD	12	Left-hippocampus	1.531 ± 0.045	0.468 ± 0.030	−0.186 ± 0.044	0.00139 ± 0.00021
		Right-hippocampus	1.519 ± 0.041	0.481 ± 0.049	−0.177 ± 0.050	0.00137 ± 0.00025

*SD, standard deviation; FM, fractional motion; ADC, apparent diffusion coefficient; AD, Alzheimer’s disease; ROIs, region of interest.*

**FIGURE 5:**
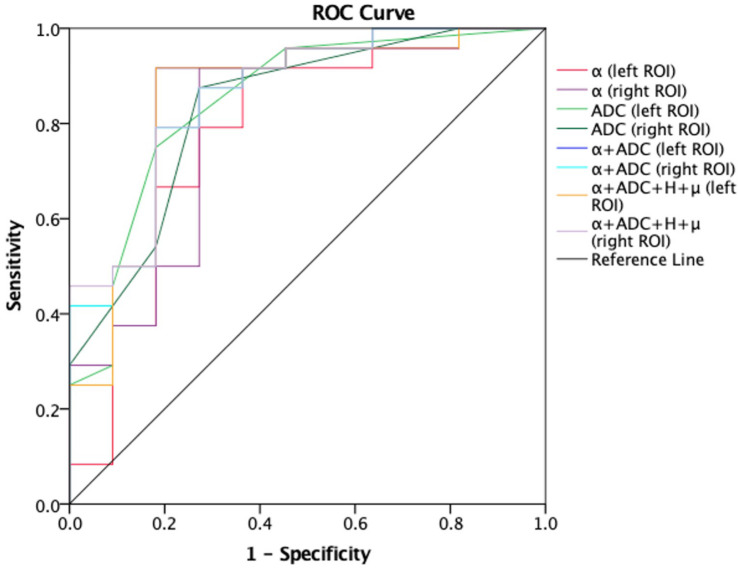
Receiver-operating characteristic (ROC) curves obtained using α, ADC, α + ADC, and α + ADC + H + μ for differentiating AD patients (*n* = 24) and healthy controls (*n* = 11). The ROC curves were generated using the mean values for subjects. From the figure we can see α + ADC (AUC = 0.848 left, AUC = 0.856 right) is superior to the single α and single ADC. α + ADC + H + μ and α + ADC have similar classification performance. ROC, reciever operating characteristic; ADC, apparent diffusion coefficient; AD, Alzheimer’s disease; AUC, area under the curve.

**FIGURE 6:**
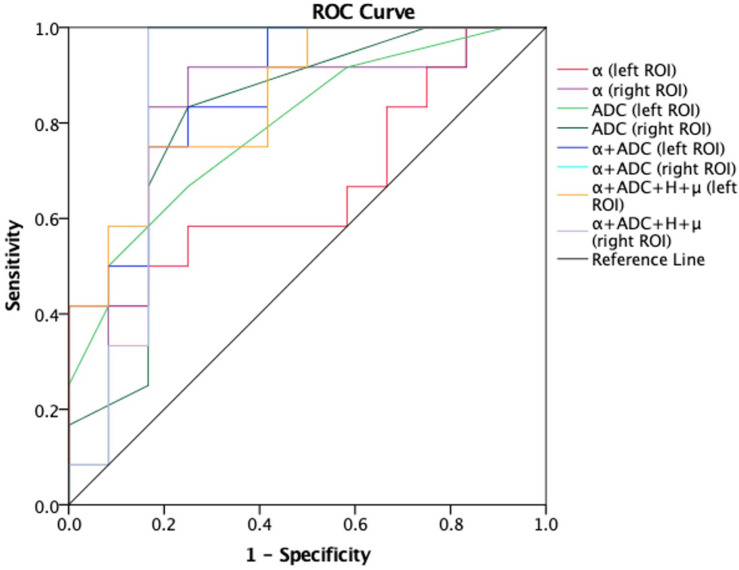
Receiver-operating characteristic (ROC) curves obtained using α, ADC, α + ADC, and α + ADC + H + μ differentiating mild AD patients (*n* = 12) and moderate AD patients (*n* = 12). The ROC curves were generated using the mean values for subjects. From the figure we can see α + ADC (AUC = 0.861 left, AUC = 0.868 right) is superior to the single α and single ADC. α + ADC + H + μ and α + ADC have similar classification performance. ROC, receiver operating characteristic; ADC, apparent diffusion coefficient; AD, Alzheimer’s disease; AUC, area under the curve.

Receiver-operating characteristic analysis also showed the performance in differentiating patients with AD from healthy controls. Figure 5 shows the ROC curves calculated from the mean values. Although the number of subjects are limited, the combination of α and ADC (AUC = 0.848 left ROI, AUC = 0.856 right ROI) showed an improved performance in differentiating AD patients and healthy controls as compared with α (AUC = 0.78 left ROI, AUC = 0.811 right ROI) or ADC (AUC = 0.847 left ROI, AUC = 0.833 right ROI) alone; when the threshold for α was 1.5939 (left ROI) and 1.58415 (right ROI), the highest Youden index (sum of sensitivity and specificity minus one) showed better sensibility and specificity (left ROI, 0.917 and 0.636; right ROI, 0.917 and 0.727). Similarly, towards differentiating mild and moderate AD patients (Figure 6), larger AUCs were obtained with α + ADC (AUC = 0.861 left ROI, AUC = 0.868 right ROI) compared with α (AUC = 0.813 right ROI) or ADC (AUC = 0.792 left ROI, AUC = 0.806 right ROI) alone; when α = 1.55115 (right ROI), the highest Youden index occurred (sensibility and specificity, right ROI, 0.917 and 0.75). The classification performance of α + ADC + *H* + μ is similar to α + ADC in differentiating AD patients and healthy controls (AUC = 0.848 left ROI, AUC = 0.860 right ROI), and grading mild and moderate AD patients (AUC = 0.847 left ROI, AUC = 0.868 right ROI).

### Correlations Between FM-Related Parameters and MMSE Scores and MoCA Scores

**FIGURE 7:**
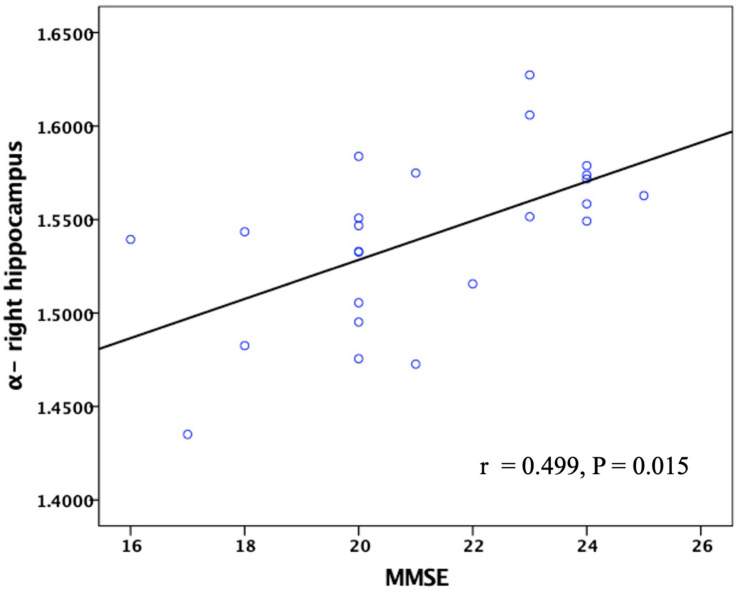
Correlation between the α values of the right hippocampus and MMSE scores in patients with AD. Pearson partial correlation was conducted, *n* = 24. MMSE, mini-mental state examination.

**FIGURE 8:**
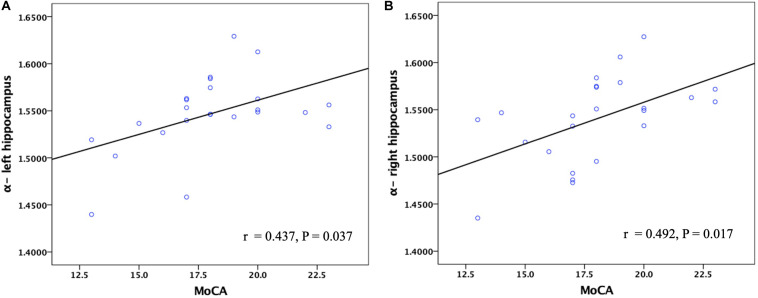
Corelation between the α values of the left and right hippocampus and MoCA scores in patients with AD. Pearson partial correlation was conducted. *n* = 24. MoCA, Montreal cognitive assessment.

The FM-related parameter α was found to be positively correlated with the MMSE score (*P* < 0.05; Figure 7) and MoCA score (*P* < 0.05; Figure 8) in patients with AD. No significant correlations were detected in other FM-related parameters and ADC. However, there was no significant correlation between α values and the MMSE score or MoCA score after false discovery rate (FDR) corrections.

## Discussion

The FM model proposed recently is a kind of anomalous diffusion model. In this study, we used the FM model to analyze anomalous diffusion in patients with AD and healthy controls in vivo. The FM-related parameter maps showed there were no obvious image contrasts visible with the naked eye in the hippocampus (Figure 2). However, the values of their anomalous diffusion parameters could be used to distinguish AD patients from healthy controls and to identify mild AD and moderate AD patients, in particular the α. The α provides better sensitivity and specificity for differentiating AD patients from healthy controls and grading mild AD and moderate AD patients. The possible explanations for this are as follows. Autopsies demonstrate the pathological changes of AD with significantly decreased numbers of neurons, atrophy of residual nerve cells, and varying degrees of degeneration. The characteristic pathological changes of AD, such as Aβ protein and neurofibrillary tangles, can eventually lead to apoptosis (Wang et al., 2015). The progression from normal brain tissue to AD is related to the development of increasing lesions, and the apoptosis and atrophy of neurons, which lead to the reduction of the hippocampal volume. The values of α depend on the structural complexity of the hippocampus, the more complex the hippocampus is, the more significant the non-Gaussian water molecule diffusion, and the greater the α values will be. In theory, the diffusion of water molecules in the brain is affected by many factors, including axons, cell membrane, and myelin (Yoshida et al., 2013). In the disease process of AD, degeneration, atrophy, and apoptosis of neurons lead to a decrease in the complexity of brain tissue, so the α values decrease in this process (Jensen and Helpern, 2010; Grinberg et al., 2011; Yoshida et al., 2013). Therefore, the α values were different between AD patients and healthy control and between mild AD and moderate AD patients.

The FM model has many advantages compared with other MRI theories. Firstly, it is more precise in describing the true diffusion signal decay curve. Secondly, the previous studies have shown that compared with ADC, FM-related parameters are more sensitive and specific in the identification of low-grade and high-grade gliomas (Xu et al., 2017b). Thirdly, the data is relatively easy to acquire and the analysis is not complex. The FM model is a potentially better dMRI technology, so we chose the FM model as the research method of AD.

The reasons why we chose the bilateral hippocampus as ROIs are as follows: (1) AD is characterized by memory loss and cognitive decline, and the hippocampus is the main brain region that is related to learning and human memory, especially long-term memory. (2) The hippocampus is one of the main brain regions that Aβ and the hyperphosphorylation of tau proteins (Glenner et al., 1984; Brion et al., 1985; Veitch et al., 2019) are overexpressed, which are the main pathological mechanisms of AD. Besides, many studies found that the internal structure of the hippocampus is asymmetric. And asymmetric expression of proteins and other molecules, and asymmetrical hippocampal morphology have been recently proposed. The functions of the left and right hippocampus are also asymmetrical, the left hippocampus is dominant in the encoding and information transfer stage, and the right hippocampus is dominant in the memory compensation stage (Song et al., 2019). So we compared them separately in this study (Acosta-Cabronero et al., 2013; Du et al., 2018).

Diffusion magnetic resonance imaging investigates the diffusion process at the cellular scale in brain tissues. Different from the mono-exponential model, FM models displayed a better agreement between the experimentally measured signal decay curve and the fitted curves (Magin et al., 2008). From Eq. 1 we can see, the α is an exponent of the diffusion gradient proportional to the parameters offered by other dMRI models, such as the stretching parameter in the stretched exponential model (Bennett et al., 2003; Hall and Barrick, 2008; Zhou et al., 2010). It is reasonable to generalize our finding of α to these parameters. Previous research has shown that the parameters calculated by this method are reliable. And the α refers to the variances of increments of diffusion processes based on the FM theory when the parameter has been interpreted as an index of heterogeneity of water diffusion (Fan and Gao, 2015). It should be admitted that the acquisition and calculation will be largely simplified if only this kind of parameters is of interest. This simplification will reduce the scan time and improve the availability. It is worth mentioning that although *H* and μ of the FM model showed no significance in this research, they do provide distinctive information and may be useful if fully explored (Xu et al., 2018).

In terms of identifying AD patients from healthy controls, the ADC values in the bilateral hippocampus of our AD patients were higher than those in healthy controls. These findings were similar to the results of other teams (Kantarci et al., 2001; Petersen, 2004; Zimny et al., 2013). We also found that the α value of our AD group was lower than that of the healthy control group. The *P*-values are close in α and traditional ADC. In a previous research, the ADC, ultra-high *b*-values ADC (ADC_uh), and diffusion kurtosis imaging (DKI) were used for AD identification. The AUC of a single parameter was 0.766–0.847, and the AUC of a combination of all three parameters was 0.868 (Xue et al., 2019). In our study, the ADC and FM model independently showed a similar ability of identification. When they were combined, the AUC value increased, indicating that the α was useful and can significantly improve the diagnostic capability of ADC when combining the α and the ADC.

Similarly, in terms of differentiating mild AD and moderate AD patients, the ADC value in the bilateral hippocampus of moderate AD patients was higher than that of mild AD patients, and the α value of moderate AD patients was lower than that of mild AD patients. Besides, the α can also significantly increase the diagnostic capability of ADC when distinguishing mild AD and moderate AD patients. Therefore, the α is very valuable in the research of differentiating and grading AD patients. In further research, the FM model combined with ADC can greatly improve the diagnostic capability of AD.

Age was an important risk factor that may affect the diffusion process of water in brain tissues and the cognitive function of AD patients. In order to eliminate the influence of age, Pearson partial correlation analysis was used to compare the relationship between the values of FM-related parameters and MMSE or MoCA scores. And we found that the α values of the hippocampus were positively correlated with MMSE and MoCA scores in the AD patients, while there were no significant correlations in ADC values. The explanation may be as follows. The values of α depend on the structural complexity of the hippocampus, the more complex the hippocampus is, the greater the α values will be. In the disease process of AD, degeneration, atrophy, and apoptosis of neurons lead to a decrease in complexity of the brain tissue (Jensen and Helpern, 2010; Grinberg et al., 2011), so the α values decrease in this process. It is well known that the cognitive function of AD patients is gradually declining. Therefore, the α values of the hippocampus were positively correlated with cognitive measures. These findings indicate that the α values of the hippocampus, better than traditional ADC values, might become a potential MRI-based biomarker for disease severity in the mild and moderate AD patients. However, there was no significant correlation between α values and the MMSE score and MoCA score after FDR correction, indicating that their correlation is not so strong. A possible reason for this was that the sample size was small.

This study has some limitations. Firstly, the number of AD patients and healthy controls in the present study is limited, so further research with a larger sample size is required to validate our results. Secondly, we only targeted patients with AD and healthy controls, the inclusion of patients with MCI in further studies is more beneficial for clinical use, since MCI is considered an early stage of AD (Petersen, 2004). Thirdly, the single-shot echo-planar imaging used in this study may lead to signal loss and image distortion. These artifacts still occur although they have been reduced due to the development of high-performance gradients and parallel imaging, so the evaluation of the hippocampus may be confined. Fourthly, the voxel was large, so a single voxel showed an aggregated measure of a large sample size, which may obstruct sensitivity to the tissue occupying a small part of a voxel. Further work is needed to explore the voxel-wise radiologic-pathologic correlation.

## Conclusion

In the present study, we selected the FM model to quantitatively calculate the FM-related parameters α, *H*, μ, and ADC values of the bilateral hippocampus. Our results showed that AD patients and healthy controls can be readily separated using the α and ADC, and mild AD and moderate AD patients can be also distinguished using the α and ADC. It was worth mentioning that the FM-related parameter α of the hippocampus was positively correlated with the cognitive function assessed by MMSE and MoCA scales in the AD patients group, while there was no correlation in ADC values, indicating that the α in the bilateral hippocampus might be a potential MRI-based biomarker of disease severity in patients with AD. This new diffusion model might be useful for further understanding neuropathologic changes in patients with AD.

## Data Availability Statement

All datasets presented in this study are included in the article/supplementary material.

## Ethics Statement

The studies involving human participants were reviewed and approved by Ethics Committee of China-Japan Friendship Hospital. The patients/participants provided their written informed consent to participate in this study. Written informed consent was obtained from the individual(s) for the publication of any potentially identifiable images or data included in this article.

## Author Contributions

LD, BX, and ZZ analyzed and explained the data and drafted and revised the manuscript. LD and GM designed the study. WG, XH, SSh, XL, YC, YW, and SSu acquired data. BX, JG, and LZ revised the manuscript about FM model theory. All authors approved the final manuscript.

## Conflict of Interest

BX was employed by company Beijing Intelligent Brain Cloud Inc. The remaining authors declare that the research was conducted in the absence of any commercial or financial relationships that could be construed as a potential conflict of interest.
